# Potential Broad-Spectrum Antiviral Agents: A Key Arsenal Against Newly Emerging and Reemerging Respiratory RNA Viruses

**DOI:** 10.3390/ijms26041481

**Published:** 2025-02-10

**Authors:** Quynh Xuan Thi Luong, Phuong Thi Hoang, Phuong Thi Ho, Ramadhani Qurrota Ayun, Taek Kyun Lee, Sukchan Lee

**Affiliations:** 1 Department of Integrative Biotechnology, Sungkyunkwan University, Suwon 16419, Republic of Korea; quynh.ltx2017@gmail.com (Q.X.T.L.); hoangphuong06cs@gmail.com (P.T.H.); hophuongk59sinhhoc@gmail.com (P.T.H.); ramadhani.qurrota@gmail.com (R.Q.A.); 2 Risk Assessment Research Center, Korea Institute of Ocean Science & Technology, Geoje 53201, Republic of Korea

**Keywords:** respiratory RNA virus, viral infections, broad-spectrum antiviral agents, direct-acting antiviral, host-directed antiviral

## Abstract

Respiratory viral infections present significant global health challenges, causing substantial morbidity and mortality, particularly among highly susceptible components of the population. The emergence of pandemics and epidemics, such as those caused by influenza viruses and coronaviruses, emphasizes the urgent need for effective antiviral therapeutics. In this review, we explore the potential of broad-spectrum antiviral agents targeting respiratory RNA viruses, including influenza viruses, coronaviruses, respiratory syncytial virus, human metapneumovirus, human parainfluenza viruses, and rhinoviruses. Various broad-spectrum direct-acting and host-targeting antivirals are discussed, including monoclonal antibodies targeting conserved regions of viral surface proteins, molecules interfering with host cell receptors or viral replication machinery, viral protease inhibitors, siRNA therapies, ribonuclease, and 3D8 scFv. Advancements in host-targeting approaches to reduce resistance and RNA-based therapeutics offer significant potential for combating respiratory viral threats. Despite challenges, broad-spectrum antiviral agents represent a crucial strategy, particularly when specific viral pathogens are unidentified or rapid intervention is essential, such as during pandemics or outbreaks.

## 1. Introduction

Respiratory viral infections pose significant global health challenges, constituting a primary cause of morbidity and mortality worldwide, particularly affecting vulnerable populations such as infants, older adults, and immunocompromised individuals [1,2,3]. In particular, the 20th century has witnessed three influenza pandemics, viz., in 1918 (associated with H1N1 with about 20–100 million deaths), 1957 (associated with H2N2 with 1.1 million deaths worldwide), and 1968 (associated with H3N2 with 1 million deaths worldwide) [4,5,6,7,8,9,10]. The emergence of the novel H1N1/pdm09 pandemic in 2009 marked the first influenza pandemic of the 21st century, causing approximately 151,700–575,400 deaths worldwide during its inaugural year of circulation [10,11,12,13]. Before the COVID-19 pandemic, lower respiratory tract infections caused 2.6 million deaths in 2019 and 3.2 million deaths in 2015 [14].

Annually, seasonal influenza accounts for billions of cases worldwide, with 3–5 million cases causing severe disease and 290,000–650,000 respiratory fatalities, especially 99% mortality in children aged <5 years with influenza infections in developing countries [15,16,17,18]. Furthermore, among seven coronaviruses capable of infecting humans, four coronaviruses (OC43, HKU1, NL63, and 229E) induce mild symptoms, whereas others (SARS-CoV-1, MERS-CoV, and SARS-CoV-2) are associated with severe syndromes [19,20]. The emergence of COVID-19 in 2019 has resulted in more than 45 million confirmed cases in 2020, and up to the time of writing this review, more than 775 million reported cases and more than 7 million deaths as of March 2024 [21,22].

Over the past two decades, respiratory RNA viruses (RRVs) have dominated recent epidemics/pandemics, including SARS-CoV, the influenza H1N1 virus in 2009, Middle East respiratory syndrome coronavirus (MERS-CoV), and the ongoing SARS-CoV-2 outbreak [21,23,24]. Several other human RNA viruses have also been implicated in upper and lower respiratory tract infections, as summarized in Table 1. In particular, RNA viruses belonging to the *paramyxoviridae* family, typically respiratory syncytial virus (RSV), human metapneumovirus (HMPV), and human parainfluenza viruses (HPIVs) types 1–3 predominantly cause bronchiolitis and pneumonia in young children [25]. RSV infections contribute to approximately 3.6 million hospitalizations and more than 100,000 deaths, and HMPV infections present a significant threat to the health of >86% of children aged <5 years [26,27,28,29]. Unlike the influenza virus or SARS-CoV-2, which have been partially controlled through vaccination programs, vaccines for RSV infections were approved only for adults in 2023 and not for children, and there is currently no vaccine on the market for HMPV infections [30,31]. There are also currently no approved antiviral drugs for infections caused by rhinoviruses (HRV), HMPV, and HPIV [31,32,33]. Consequently, RRVs have the potential to induce severe respiratory illnesses and impose substantial socioeconomic and healthcare burdens.

RRVs possess negative-sense or positive-sense RNA, exhibit higher mutation rates, rapid replication cycles, and the ability to adapt and evolve rapidly, thereby bypassing or avoiding the immunogenic response of host cells and increasing the risk of antiviral drug resistance [24,34,35]. Therefore, there exists an urgent need for effective therapeutics and prophylaxis targeting RRVs to combat infectious diseases. Nevertheless, the continuous evolution of RNA viruses, coupled with the surgency or resurgence of viruses and the development of antiviral drug resistance, presents additional challenges for already strained healthcare systems in response to epidemics or pandemics, as exemplified by the ongoing COVID-19 pandemic [36,37,38]. Hence, the development of drugs with broad-spectrum antiviral activities is imperative [24,39]. In this review, we explore promising candidates that have demonstrated broad-spectrum antiviral activity against various respiratory viruses, focusing on both direct-acting antivirals and host-targeted antivirals at different stages of the viral life cycle. We assess their mechanisms of action, evaluate the challenges of drug resistance, and reflect on key lessons learned from the COVID-19 pandemic to inform future strategies for combating respiratory viral threats.

**Table 1:** Overview of RRVs and their impact on human health.

Virus	Family	RNA Genome	Replication Location	Major Clinical Disease	The Prevalence of RRVs	Available Therapies
Coronavirus (CoV-229-E, CoV-OC43, CoV-NL63, CoV-HKU1, MERS-CoV, SARS-CoV, SARS-CoV-2)	*Coronaviridae*	Ss (+)	Cytoplasm	Upper and lower respiratory tract. SARS-CoV-2, MERS-CoV can trigger respiratory failure, significant mortality in older adults, patients with cardiovascular, respiratory disorders. The others cause the mild symptoms [20,40]	COVID-19: 775 million cases, more than 7 million deaths [22]. In the US, OC43, HKU1, and NL63 caused 3%, 2.6%, and 2.4% infections in December and January 2023, respectively. At the same time of 2024, the proportions were 2.9%, 1.9%, and 1%, respectively. However, the infections caused by the 229-E strain were nonsignificant [20]	Antiviral medications: Nirmatrelvir/Ritonavir (Paxlovid), Veklury (Remdesivir), Molnupiravir (Lagevrio), Hydroxychloroquine/Chloroquine + Azithromycine, Lopinavir/Ritonavir. Neutralzing antibodies for pre-exposure prophylaxis (Pemivibart) Immunosuppressive therapies Interleukin-6 inhibitors, Glucocorticoids, Corticosteroids, Janus kinase inhibitors Covalescent plasma [41,42] For common human coronaviruses: There is no specific antiviral therapy to treat common human coronaviruses [20,41] For MERS-CoV: There is no specific antiviral therapy to treat MERS-CoV [43,44]
Influenza virus (IAV, IBV)	*Orthomyxoviridae*	Ss (−)	Nucleus	Upper respiratory tract. Both type A and B spread in people and are responsible for seasonal flu annually [45]	3–5 million cases of severe illness 290,000–650,000 deaths annually 99% deaths in children aged <5 years [18]	Antiviral treatments Oral oseltamivir, oral Baloxavir, inhaled Zanamivir, intravenous Peramivir [46,47]
Metapneumovirus (HMPV)	*Pneumoviridae*	Ss (−)	Cytoplasm	Upper and lower respiratory tracts infection. Acute respiratory tract disease in children, older adults, and immunocompromised patients, especially in infants (aged <5 year), causing coughing, wheezing, fever, bronchiolitis, and pneumonia [48,49,50]	The percentage of HMPV-positive tests in the US fluctuated from 5% to 12–13% within a year (April 2023–April 2024) [50]	There is no specific antiviral therapy to treat HMPV, only supportive care [51].
Parainfluenza virus (HPIV)	*Paramyxoviridae*	Ss (−)	Cytoplasm	Upper and lower respiratory tract infections. Affects children, causing bronchiolitis and pneumonia. HPIV3 primarily affects young infants. HPIV1 and 2 tend to infect older children and adolescents [25]	2,700,135 HPIV tests were reported. 122,852 (5%) were positive for HPIV, including 22,446 for HPIV-1 (18%); 17,474 for HPIV-2 (14%); 67,649 for HPIV-3 (55%) and 15,284 for HPIV-4 (13%) (reported in July 2011–June 2019) [52]	There is no specific antiviral therapy to treat HPIV, only supportive care [53].
Respiratory syncytial virus (HRSV)	*Pneumoviridae*	Ss (−)	Cytoplasm	Lower respiratory tract infection. Primarily affects small children causing obstructive bronchiolitis, resembling bronchial asthma [54]	In 2019, 3.6 million hospitalizations; more than 100,000 deaths [29]. In the US, the percentage of RSV-positive tests reached approximately 20% in October and November 2023 and approximately 12.5% at the same time of 2024 [55]	There is no specific antiviral therapy to treat HRSV, only supportive care [56,57,58].
Rhinoviruses (HRV)	*Picornaviridae*	Ss (+)	Cytoplasm	Upper and lower airway infections. Cause of common cold and a major trigger for exacerbations of lower respiratory tract diseases [59]	19.29%, 22.1%, and 1.32% of HRV infections (including enterovirus) were detected in children aged <2 years with bronchiolitis, children with community-acquired pneumonia, and children and adults with COVID-19 [60,61,62,63].	There is no specific antiviral therapy to treat HRV, only supportive care [64].

## 2. Approaches for Developing Broad-Spectrum Antiviral Therapeutics Based on Viral Replication Cycle

### 2.1. Viral Attachment Inhibitors

The process of viral attachment marks the initial step in which viruses gain entry into cells through the interaction between viral surface proteins and cell receptors. Preventing this interaction represents a promising approach in the development of antiviral agents [65,66]. Thus, viruses can be inhibited by blocking viral protein-receptor binding using antibodies or small molecule inhibitors.

Hemagglutinin (HA) is obviously the most prevalent surface glycoprotein, with the HA stalk domain remaining remarkably conserved among influenza viruses due to functional constraints and limited immune pressure [67]. Therefore, the development of potential broad-spectrum monoclonal antibodies as direct-acting antiviral agents involves blocking the receptor-binding site by binding to the conserved stem region in HA protein and inhibiting HA-mediated viral fusion between endosomal and viral membranes [68,69]. CR6261, a monoclonal anti-HA stalk antibody, exhibits broad neutralizing activity [70,71]. It neutralizes various influenza subtypes, including H1, H2, H5, H6, H8, and H9, and provides protection against murine lethal challenge models with H1N1 and H5N1 viruses [70,71,72]. During viral maturation, HA polypeptide undergoes proteolytic cleavage into two disulfide-linked subunits, viz., HA1, which binds sialic acid receptors, and HA2, responsible for the fusion step [71,73]. The antibody neutralizes the virus by impeding the pH-induced conformational changes in HA associated with membrane fusion [71,72,74,75]. Cocrystal structures of CR6261 Fab in complexes with the HA of 1918 H1N1 (A/South Carolina/1/1918) and H5N1 (A/Vietnam/1203/2004) have been clarified. Phase 1 trials (NCT01406418) of CR6261 have demonstrated safety, although the effective prevention of influenza infection may be limited in phase 2 trials (NCT02371668) [70]. It has been reported that oseltamivir/zanamivir may improve the therapeutic efficacy of CR6261 [76]. Furthermore, other cross-reactive monoclonal anti-HA stalk antibodies have been evaluated in phase 1 or 2 clinical trials, such as MEDI8852, VIS410, and CT149. These antibodies exhibit binding to a highly conserved epitope on the HA of the influenza virus as observed by crystallographic analysis, demonstrating efficacy for prophylaxis and treatment for group 1 (H1, H2, H5, H6, H8, H9, H11, H12, H13, H16,H17, and H18) and/or group 2 (H3, H4, H7, H10, H14, and H15) influenza virus infections in animal-based assays [67,68,69,77,78,79]. Despite the broad neutralization potential of anti-HA stalk antibodies, several viral mutations have been shown to impair their binding and functionality [80,81,82]. Notably, the HA mutation A388V induces a conformational change in the stalk region, disrupting key epitopes targeted by various broadly neutralizing antibodies [82]. This mutation has been demonstrated to significantly reduce the binding affinity of multiple well-characterized bNAbs, including CR6261, CR9114, FI6V3, 70-1F02, C179, and CT149, compared to wild-type HA [82]. Additionally, head mutations that confer resistance to strain-specific antibodies may also allow the virus to escape broadly neutralizing antibodies targeting conserved stem regions [80]. In addition, they face significant limitations as well. Production costs are high, potentially limiting widespread accessibility [83]. Furthermore, clinical trials have reported adverse events with some antibodies, such as VIS410, which have been associated with vomiting and diarrhea [84]. The efficacy of therapies like CR6261 may also be limited when used as standalone treatments, and it may achieve greater success when employed in combination with other antiviral strategies, rather than as sole therapeutic agents or vaccines [70].

In addition to targeting HA to prevent the attachment of viruses, targeting host sialic acids is another approach. Typically, DAS181, a host-directed antiviral, comprises a recombinant construct containing a heparin-binding domain and the catalytic domain of *Actinomyces viscosus* sialidase. It effectively cleaves α2,3- and α2,6-linked sialic acid receptors on the cell surface, indirectly inhibiting the viruses that bind to host cells via sialic acid receptors [65,68,85]. Because of its host-targeting mechanism of action, it indicates that resistance to DAS181 is low and unstable [86]. DAS181 has exhibited antiviral activity against influenza A (H1N1, H3N2, H5N1, and oseltamivir-resistant H1N1) and B viruses and PIV in cell-based assays and animal models by removing both types of sialic acid in birds and humans [68,86,87,88,89]. Moreover, ongoing clinical phase 3 trials are investigating its ability to improve oxygenation in select severely immunocompromised patients with PIV (NCT0164487), following successful completion of phase 2 trials (NCT01037205) with seasonal H3N2, pandemic 2009 H1N1, and influenza B virus [88,90,91].

Umifenovir (Arbidol), a broad-spectrum antiviral drug developed by a Russian company and has been approved for the prophylaxis and treatment of respiratory viral infections (including influenza A and B viruses) in Russia (1993) and China (2006) but not in North America [92,93]. Umifenovir demonstrated antiviral activity against various RRVs, including influenza A, B, and C, RSV, SARS-CoV, HCoV-OC43, HCoV-229E, SARS-CoV-2, and PIV-5 in vitro and/or in vivo [93,94,95,96,97]. Umifenovir is an indole-based hydrophobic molecule and can form supramolecular structures by interacting with specific aromatic residues within viral glycoproteins, critical for membrane interactions and instability required for fusion, and with viral and/or cellular proteins, thereby blocking viral endocytosis and replication [93,94]. Thus, umifenovir exhibits features of both a direct-acting antiviral and a host-targeting agent [94]. It has been reported to be safe and well tolerated in humans [93], and its effectiveness has also been evaluated in patients with influenza, acute respiratory viral infections, and SARS-CoV-2, demonstrating efficacy found in patients infected with respiratory viruses [92,98,99,100]. Umifenovir was found to be effective against influenza viruses in the clinical trial phase 4 conducted during the 2011–2016 influenza seasons (NCT0165663) [101]. However, some meta-analyses of umifenovir therapy for acute respiratory viral infections have yielded varied results, demonstrating its marked efficacy against influenza virus but inconclusive evidence concerning its effectiveness against respiratory viral infections caused by coronaviruses and its effect on the clinical outcomes of COVID-19 [102,103].

Aprotinin (APR), a pan-protease inhibitor commonly used in high-risk patients undergoing cardiopulmonary bypass surgery to reduce bleeding, was identified as a potential broad-spectrum antiviral candidate against RRV infections due to its capacity to inhibit the proteolytic activation of some viruses [104,105,106,107]. IAVs and SARS-CoV-2 have evolved a two-step activation process requiring the proteolytic cleavage of HA for IAVs and spike glycoprotein for SARS-CoV-2 into HA1, HA2, and S1, S2, respectively [107,108]. The cleavage of HA in influenza viruses is crucial for virus—host fusion, because influenza viruses cannot initiate infection unless HA is proteolytically cleaved by a trypsin-like protease, whereas the cleavage of spike protein plays a vital role in viral entry and replication, occurring at a furin-like domain by proteases such as trypsin, and kallikrein, members of the TMPRSS family of serine proteases expressed in human bronchial epithelial cells [107,108,109,110]. APR effectively targets TMPRSS2, responsible for the entry of SARS-CoV-2 into cells, and trypsin-like proteases responsible for cleaving the HA of influenza virus [106,107,110]. Studies have suggested that APR exerts significant inhibitory effects in vitro against IAVs and coronaviruses, including seasonal human IAVs (H1N1, H3N2), avian IAVs (H5N2, H6N5, and H9N2), an oseltamivir-resistant IAV, influenza B virus, and SARS-CoV-2 [104,107,109,111,112]. Recently, a phase 2 clinical trial investigated APR against COVID-19, demonstrating its efficacy and safety in patients with moderate COVID-19 by nebulization [108,109].

Recently, a monoclonal antibody, 3E8, was generated to prevent the entry of all ACE2-dependent coronaviruses to cells, including hCoV-NL63, SARS-CoV, SARS-CoV-2, and SARS-CoV-2 mutant variants (SARS-CoV-2-D614G, B.1.1.7, B.1.351, B.1.617.1, and P.1) [113]. By targeting the RBD-binding site on human angiotensin-converting enzyme 2 (ACE2), 3E8 particularly blocks the S1-subunits and pseudo-typed virus constructs [113]. Another research team reported the generation of six human monoclonal antibodies that showed similar results to 3E8 with preventing SARS-CoV-2, Delta, and Omicron variants by targeting the ACE2 [114]. Both teams demonstrated that these targeting ACE2-monoclonal antibodies did not cause severe toxicity to ACE2 knock-in mice and significantly impacted the enzymatic activities of ACE2 [113,114].

### 2.2. Fusion Inhibitors

Viral entry concludes when the virus reaches the cytosol following endocytic uptake, utilizing diverse initial trafficking pathways to the site of membrane fusion. Disruption of the endo-/lysosomal trafficking by fusion inhibitors may sequester viral particles within vesicles, thereby hindering fusion with the endosomal limiting membrane and causing subsequent release of viral genome into the cytosol [115].

**Figure 1:**
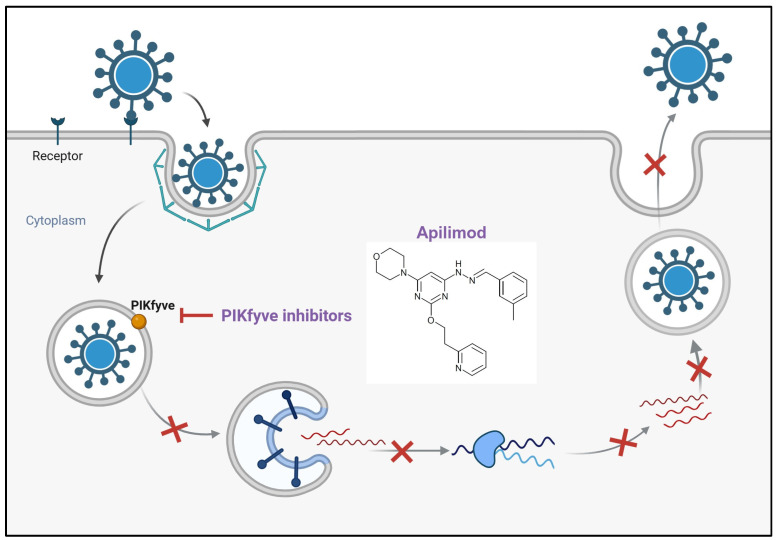
Targeting PIKfyve for antiviral therapy involves disrupting its role in endosomal trafficking. Inhibiting PIKfyve activity interferes with the maturation and function of endolysosomes, blocking the escape of endocytosed viruses into the cytoplasm. This effectively halts the viral replication process by preventing the release of viral genetic material required for infection.

PIKfyve (phosphatidylinositol-3-phosphate 5-kinase type III) plays a pivotal role in regulating endomembrane homeostasis [116,117,118]. Inhibition of PIKfyve results in the enlargement of endosomes into small, spherical vacuoles [115]. Consequently, viral particles are sequestered within these vacuoles adjacent to the endosomal membrane, preventing fusion, leading to the inhibition the release of the viral genome into the cytosol (Figure 1) [115,119,120]. PIKfyve inhibitors such as apilimod and XMU-MP-7, have demonstrated efficacy in suppressing the replication of SARS-CoV-2 and its variants (alpha, beta, delta, and omicron) in cell-based assays, and phase 2 clinical trials are also investigating apilimod against COVID-19 (NCT04446377) [116,118]. Nevertheless, the effectiveness observed in vitro does not always translate to that observed in vivo, as evidenced by a COVID-19 murine model study that investigated both prophylactic and therapeutic interventions [116]. In this study, the effectiveness of antivirals observed in vitro fails to replicate in vivo due to the significant differences in complexity between these systems, bioavailability, host–pathogen interactions, and model limitations [116]. Particularly, the inhibitors suppress proinflammatory cytokine secretion, impairing immune cell recruitment and trafficking [116]. This immune modulation delays viral clearance, leading to worse disease outcomes [116]. Single-cell analysis reveals decreased expression of interferon-stimulated genes despite elevated viral loads, indicating that PIKfyve inhibition disrupts innate immune responses, compromising the body’s ability to control viral replication [116]. Moreover, apilimod has been demonstrated to inhibit the cytopathic effect induced by H1N1, H3N2, H5N1, and influenza B viruses with IC50 values ranging from 3.8 to 24.6 µM, along with substantial reductions in viral load, prevention of weight loss, and attenuation of inflammation in influenza virus-infected mouse models [121]. Another study demonstrated that apilimod exerts antiviral effects against RSV in human nasal epithelium in vitro and in mouse models in vivo [121]. These inhibitors demonstrated no significant impact on cell viability at concentrations exceeding 40 µM for apilimod and 150 µM for XMU-MP-7, indicating a wide safety margin concerning mammalian cell toxicity [118,121].

Peptide P9 and its mutant P9R, derived from mouse b-defensin-4, have been identified as broad-spectrum antiviral agents targeting both host cells and viruses [122]. These peptides exhibited potent antiviral activity against multiple respiratory viruses, including SARS-CoV, MERS-CoV, SARS-CoV-2, pandemic H1N1, H3N2, H5N1, H7N7, H7N9, and HRV in vitro [122,123]. The antiviral effects were significantly improved by enhancing the net positive charge from (+4.7) of P9 to (+5.6) of P9R through the substitution of weakly positively charged residues with arginine residues [122]. Considering that endosomal acidification is regulated by the influx of protons into the endosome, an alkaline peptide with a higher net positive charge could reduce proton concentrations within the endosome, potentially hindering virus–host endosomal acidification and blocking the endosomal release of pH-dependent viruses [122,124]. It has been shown that P9R not only binds to viruses but also inhibits endosomal acidification [122]. Moreover, P9R effectively protects mice from H1N1/pdm09 challenge and does not induce the formation of drug-resistant viruses after 40 passages of simultaneous co-culture with P9R [122].

### 2.3. Viral Biosynthesis Inhibitors

#### 2.3.1. Viral Protease Inhibitors

Viral proteases are enzymes encoded by the genome of some viruses with catalytic activity capable of hydrolyzing peptide bonds at specific locations within polyprotein chains (Table 2) [125]. Several viruses encode one or more proteases as a fundamental tactic, as these proteases play a critical role in the life cycle of viruses to produce mature viral proteins by cleaving the polyprotein precursors at several distinct locations to functional products [126,127]. The amino acid sequences of the cleavage sites recognized by specific viral proteases are typically diverse and undergo processing at varying rates [126].

Viral proteases utilize diverse catalytic mechanisms involving aspartic acid, cysteine, threonine, or serine residues to target the scissile peptide bond frequently found within conserved sequence motifs of up to 10 residues in length [125]. Viral proteases represent prime targets for therapeutic intervention due to their indispensability for viral replication.

Coronaviruses harbor (+) ss-RNA genome that encodes two large replicase polyproteins, cleaved by two viral-encoded cysteine proteases, viz, 3CL protease (3CLpro) and papain-like protease (PLpro), yielding nonstructural proteins such as RNA-dependent RNA polymerase (RdRp) and helicase [128,129,130]. Several PLpro inhibitors exhibit narrow-spectrum activity because of structural differences among the PLpro of different coronaviruses, exemplified by the structural dissimilarities in flexible blocking loop 2 domains between SARS-CoV and MERS-CoV and MERS-CoV PLpro, rendering MERS-CoV unaffected by the PLpro inhibitors of SARS-CoV [130,131,132]. Conversely, drugs designed to target the 3CLpro enzyme hold promise as broad-spectrum antivirals due to its high conservation [130,133]. Kaletra, a combination of lopinavir–ritonavir, approved for HIV treatment, failed to reduce hospital stays or mortality rates in patients with COVID-19 within 28 days, according to clinical trials conducted by the United Kingdom Recovery and Lotus China [134,135,136]. The variations in substrate-binding sites between HIV and SARS-CoV-2 proteases may be a key to understanding why some antiviral protease inhibitors, such as Kaletra (lopinavir/ritonavir), are ineffective against COVID-19. HIV protease is a C2-symmetric homodimeric aspartyl protease, while SARS-CoV-2’s main protease (Mpro) is a cysteine protease [134]. These proteases have distinct substrate specificities and binding pocket geometries; SARS-CoV-2 3CLpro prefers substrates with a glutamine at the P1 position, whereas HIV protease targets the interface between the two monomers and contains the catalytic Asp-Thr-Gly residues for viral polyprotein cleavage [134,137,138,139]. These differences significantly limit the cross-application of inhibitors designed for HIV. Nevertheless, lopinavir demonstrated efficacy against SARS-CoV in vitro by inhibiting the 3CLpro enzyme and exhibiting antiviral activity when combined with ritonavir [140,141,142,143]. Lopinavir also inhibited the MERS-CoV-induced cytopathic effects in vitro and reduced viral loads, improved pulmonary function, reduced lung hemorrhage, and attenuated weight loss when used prophylactically in combination with IFN-β in animal models [140,144,145,146].

Paxlovid, a combination of nirmatrelvir and ritonavir, was approved for SARS-CoV-2 treatment by the FDA, in which nirmatrelvir has shown broad-spectrum antiviral activity against SARS-CoV-2, MERS-CoV, hCoV-229E, hCoV-NL63, and hCoV-OC43 by binding to the catalytic site cysteine, resulting in blocking the function of 3CLpro [147,148,149,150]. Simultaneously, Paxlovid demonstrated an 89% reduction in the risk of hospitalization or death in high-risk patients during phase 3 trials when administered within three days of symptom onset [151]. This efficacy was slightly lower (85%) when administered within five days. Additionally, Paxlovid reduced viral load significantly at Day 5, reinforcing its robust antiviral activity across various SARS-CoV-2 variants, including Omicron [151]. The trials also reported fewer adverse events compared to placebo, with most side effects being mild (fda.gov) [151,152]. These findings have established Paxlovid as a key oral antiviral, significantly reducing severe COVID-19 outcomes.

Several highly potent inhibitors of 3CLpro with broad-spectrum activity against pan-coronaviruses have been recently developed. Various small molecule analogs of GC376 (EB46, EB54, and NK01-63) were found to inhibit 3CLpro and suppress the replication of SARS-CoV, SARS-CoV-2, MERS-CoV, HCoV-229E, and HCoV-OC43, with EC50 values ranging from 0.1 to 2.4 µM in cell-based inhibition assays [129]. Cyclopropane-based inhibitors, including aldehydes 5d and 11d, were found to inhibit the 3CLpro of MERS-CoV and SARS-CoV, with IC50 values of 70–70 and 790–240 nM, respectively, and aldehydes 5c and 11c were also found to inhibit the replication of SARS-CoV-2, with EC50 values between 12 and 11 nM [128]. Another study showed that compounds C2–C5a, which belong to a novel class of active-site-directed 3CLpro inhibitors, demonstrated broad-spectrum activity against omicron subvariants (BA.5, BQ.1.1, and XBB.1.5) and HCoV-229E in human cells [153]. The contrasting outcomes between Kaletra and Paxlovid underscore the critical role of designing inhibitors tailored to the substrate-binding specificity of SARS-CoV-2’s main protease. The success of Paxlovid is attributed to its precise targeting of this protease, crucial for viral replication, highlighting a more promising pathway for antiviral development.

Considering the indispensability of RNA polymerase for the replication of RNA viruses, a specific inhibitor of viral RNA polymerase would exhibit antiviral activity without affecting mRNA synthesis or protein translation in host cells [65]. Galidesivir (BCX4430) is a direct-acting antiviral compound, an adenosine analog, and an RdRp inhibitor, interrupting the synthesis of viral RNA [154]. Nucleoside analogs mimic the structure of natural purine or pyrimidine nucleosides by modifying the base or ribose sugar moieties, or both, to be recognized by viral RdRp [154,155]. In the case of BCX4430, with two substitutions at position 7 on the adenosine ring (carbon to nitrogen) and position 1 on the ribose ring (nitrogen to oxygen), it alters to an azasugar ring, preventing the addition of more nucleotides by viral RdRp, resulting in the disruption of viral propagation [154,155]. BCX4330 exhibited antiviral activity against numerous RNA viral pathogens in vitro, including RRVs such as MERS-CoV, SARS-CoV, IAV, RSV, and HRV, with EC50 values of 68.4, 57.7, 10.7, 11, and 3.4 µM, respectively [154,155,156]. Several phase 1 clinical trials are currently investigating the efficacy of BCX4330 against viruses, including SARS-CoV-2 [154,155].

During viral gene expression and replication in virus-infected cells, the de novo pyrimidine biosynthesis pathway is activated to fulfill the high demand for pyrimidines [54,157,158]. Thereafter, a mitochondrial enzyme, human dihydroorotate dehydrogenase (hDHODH), facilitates the conversion of dihydroorotic acid into orotic acid, a critical step in the biosynthesis of uridine and cytidine [54,159,160,161]. Some studies have focused on this pathway to design a small molecule hDHODH inhibitor, MEDS433, that interacts with the ubiquinone-binding site of hDHODH, effectively suppressing the replication of various RRVs, e.g., influenza A and B viruses, RSV, as well as HCoV-299E, OC43, and SARS-CoV-2 [54,161,162].

#### 2.3.2. Targeting Viral Genomes

Targeting viral genomes to develop antiviral drugs represents a promising prophylactic and therapeutic approach with broad applicability against multiple viruses. RNases, RNAi, and 3D8 scFv are among the potential antiviral candidates that can extend their inhibitory effects to various RRVs.

RNases target viral pathogens in a multifaceted strategy through direct or indirect mechanisms, including the enzymatic degradation of viral RNA, activation of interferon (IFN) pathways, induction of apoptosis, and regulation of stress granule formation [23,163]. Since 1968, researchers have observed heightened RNA-catalytic activity in the blood and cerebrospinal fluid of patients infected with tick-borne encephalitis, resulting in increased interest in RNases with documented antiviral properties [163,164]. Several RNases have demonstrated antiviral activity against some RRVs in various studies. In particular, a recombinant eosinophil-derived neurotoxin (EDN/RNases2) was found to exert inhibitory effects against both RSV-B and PIV via ribonuclease-dependent activity in cell-based assays [165,166]. RNase L also exerted antiviral effects against IAV and RSV by digesting the viral RNA in the absence of viral resistance NS1 protein and via IFN-γ-mediated inhibition in human epithelial cells, respectively [167,168]. Nonetheless, viruses have evolved mechanisms to evade the OAS/RNase L system; for instance, 2′,5′-phosphodiesterases that cleave 2–5A are released by coronaviruses and thus block OAS signaling [163,169]. Moreover, some studies have reported that binase inhibits the replication of MERS-CoV, HCoV-229E, H1N1pdm09, and HRV-1A by directly targeting the viral mRNA in vitro [170,171].

RNA interference therapy has been considered an attractive approach for combating RRVs, with small interfering RNAs (siRNAs) being the most commonly used agents [23,172]. siRNAs, short dsRNAs (21–23 bp), which are encapsulated within nanoparticles, are taken up by cells via endosomes. After escaping from endosomes, siRNAs are released into the cytosol, where the guide, or the antisense strand of siRNAs, associates with the RNA-induced silencing complex (RISC), guiding the RISC to recognize and cleave targetable regions [172]. Studies have demonstrated the efficacy of siRNAs against different RRVs, such as multiple variants of SARS-CoV-2, SARS-CoV, MERS-CoV, RSV, PIV, and influenza virus in vitro and/or in vivo and/or ex vivo [173,174,175,176,177,178,179,180,181]. Moreover, some siRNA-based antiviral drugs have progressed to clinical trials; for instance, ALN-RSV01, targeting the nucleocapsid gene of RSV, exerted antiviral effects in vitro and in vivo, and in a phase 2a trial, it reduced the RSV load in the lung of infected patients [182,183,184,185]. However, its phase 2b clinical study, which focused on lung transplant patients, did not achieve its primary endpoint of significantly reducing new or progressive bronchiolitis obliterans syndrome (BOS) in an intent-to-treat analysis [186,187]. Nevertheless, secondary analyses suggested potential benefits, such as reduced BOS incidence when treatment was initiated early. Besides that, the therapeutic use of siRNA faces significant challenges in safety, delivery, and resistance [186,187].

Additionally, the specificity of siRNAs, designed to target particular viral RNA sequences, might initially appear to limit their application to a single virus. However, under certain conditions, siRNAs can function as broad-spectrum antiviral agents. Many viruses within the same family share highly conserved RNA sequences essential for their replication or structural integrity. By designing siRNAs to target these conserved regions, they can effectively inhibit multiple strains or even different viruses within the same family. For instance, studies have demonstrated that siRNA-NP1496, which targets the conserved NP gene, not only reduces viral load but also provides protection against diverse influenza strains, including H1N1, H5N1, H6N2, H7N7, H8N4, and H9N2 [188,189,190,191,192]. Similarly, siRNA-C6G25S, targeting the conserved RdRp gene, has proven highly effective in suppressing multiple strains of SARS-CoV-2 [175]. Another approach involves targeting host factors that are essential for viral replication. Host-directed siRNAs can impede the replication of multiple, unrelated viruses that rely on the same host machinery. For example, siRNA-siA1, which targets the ACE2 receptor and has shown high efficacy in inhibiting SARS-CoV-2 infection in vitro [193].

Key hurdles include extracellular degradation, rapid clearance, and endosomal entrapment, all of which reduce efficacy [172]. For respiratory viruses, local delivery methods like intranasal or pulmonary routes can bypass some systemic obstacles [172]. However, siRNAs still require optimized formulations to ensure stability, efficient cellular uptake, and endosomal escape [194]. Addressing these challenges involves sequence modulation, chemical modifications of nucleotides, and innovative delivery systems [172,194,195,196,197,198]. Additionally, off-target effects, immune activation by dsRNA sensors, and toxicity remain significant concerns [172]. Future strategies must balance efficacy and safety, focusing on precise siRNA design and advanced delivery technologies.

The 3D8 single-chain variable fragment (3D8 scFv), a monoclonal antibody derived from an autoimmune-prone mouse model (MRL-lpr/lpr), has received attention as a potential antiviral agent [199]. Recombinant 3D8 scFv was generated by connecting the heavy chain variable single domain (VH) with the light chain variable single domain (VL) via a flexible linker ([Glycine4-Serine]3) [199]. Although the precise mechanism of action of 3D8 scFv remains unclear, it is hypothesized to directly target the viral genome because of its enzymatic activity against both DNA and RNA in the presence of Mg^2+^ [23,199,200,201]. 3D8 scFv can be produced from various systems while retaining its functional capabilities, including expression in *Escherichia coli* and *Lactobacillus paracasei*, or via planta transformation in vegetatively reproductive *Kalanchoe pinnata* [199,202,203,204]. Furthermore, 3D8 scFv can penetrate cells through caveolae/lipid raft endocytosis mediated by heparan sulfate proteoglycans and chondroitin sulfate proteoglycans, which act as endocytic receptors on the cell surface, and internalize in the cytosol without further trafficking to the nucleus or other organelles, thereby distinguishing it from other cell-penetrating anti-DNA antibodies [205,206]. With these features, 3D8 scFv has exerted inhibitory effects against a broad-spectrum of DNA and RNA viruses wherein RRVs have also been targeted, for instance, the influenza viruses H1N1/PR8, H1N1/NWS33, H1N1/pdm09, H9N2, and H3N2 and the coronaviruses HCoV-OC43, SARS-CoV-2, and PEDV in vitro and/or in vivo [23,39,200,201,207].

### 2.4. Viral Assembly and Release Inhibitors

Among the FDA-approved antivirals currently being used for the treatment of influenza virus, oseltamivir, zanamivir, and peramivir are the drugs targeting Neuraminidase (NA) enzyme, which cleaves sialic acid from the cell surface, and new virions then help the virus release from infected cells [208,209]. These antivirals are against both IAV and IBV by blocking the NA active site, which is highly conserved, resulting in impairing virus release and effectively limiting reinfection [208,209,210,211,212]. Like other antiviral drugs, NA inhibitors also faced the challenge of drug-resistant mutations in the target enzyme with the H275Y mutation. Leading to oseltamivir is no longer effective in the pandemic H1N1 treatment [209,213]. However, the H275Y mutant remains susceptible to zanamivir [213].

## 3. Positives and Negatives of Broad-Spectrum Antivirals

Broad-spectrum antiviral drugs offer versatility by targeting multiple viruses and genotypes. These drugs can serve as a first-line treatment for patients with viral infection symptoms when the responsible virus is unidentified or rapid diagnosis is unavailable [214]. Furthermore, during pandemics or epidemics caused by novel viruses, broad-spectrum antivirals can provide a vital initial line of defense, optimizing the time needed to develop specific treatments. They may also mitigate the development of resistance by targeting the host, potentially reducing treatment complexity and drug–drug interactions [215,216,217].

Nevertheless, developing broad-spectrum antiviral drugs presents challenges, including the identification of targets shared among multiple viruses while minimizing adverse effects on host cells. The risk of off-target effects introduces the possibility of unintended side effects or toxicity. Moreover, although broad-spectrum antiviral drugs may exhibit potent activity in cell-based assays, their in vivo efficacy may fluctuate [215]. In addition, host-targeting antiviral drugs carry a remarkable risk of cellular toxicity, adding another layer of complexity to their development [214]. Mutations in viral targets can reduce the efficacy of broad-spectrum antiviral drugs, posing a significant challenge that must be addressed during their development and screening. In the case of influenza viruses, 100% of circulating H1N1 and H3N2 strains have developed resistance to adamantanes, which target the M2 ion channel, while NA inhibitor oseltamivir is no longer effective in treating the pandemic H1N1 [36]. Recently, a group of researchers developed a vesicular stomatitis virus (VSV)-based system, where the 3CLpro of SARS-CoV-2 was required for VSV replication. This system was used to identify the mutations that confer resistance to nirmaltrelvir. The findings revealed that some mutants exhibited cross-resistance to other 3CLpro inhibitors, such as ensitrelvir and GC376, while others were less resistant. Moreover, many of these resistance mutations had already been identified in SARS-CoV-2 sequences deposited in the NCBI and GISAID databases, indicating their presence in circulating SARS-CoV-2 strains. Therefore, the emergence of resistance mutations in viral targets presents a major obstacle to the effectiveness of broad-spectrum antiviral drugs. The widespread resistance of influenza strains and the identification of cross-resistance mutations to multiple 3CLpro inhibitors in SARS-CoV-2 further underscore the importance of continuous monitoring of viral evolution and the need for approaching other strategies and developing next-generation antivirals capable of overcoming these resistance mechanisms. Double-, triple-combination antiviral drug treatment, and combined antiviral-immunomodulator therapy are the potential therapeutics with different modes of action that would enhance the antiviral potency and reduce the risk of resistance [209,218]. This strategy has proven highly effective in treating diseases such as HIV and hepatitis C and is increasingly being explored for other viral infections, including influenza, coronaviruses, and other RNA viruses [218,219,220,221,222,223]. The combination of antiviral drugs also presents some challenges such as increased risk of drug–drug interactions, overlapping side effects, and sometime combine therapy is less efficacy than monotherapy which was suggested by using oseltamivir and zanamivir for influenza treatment [224,225,226]. Despite these challenges, broad-spectrum antiviral agents remain indispensable in combating viral infections, particularly in scenarios where the specific viral pathogen is unidentified or rapid intervention is imperative, such as during pandemics or outbreaks.

## 4. Conclusions

**Figure 2:**
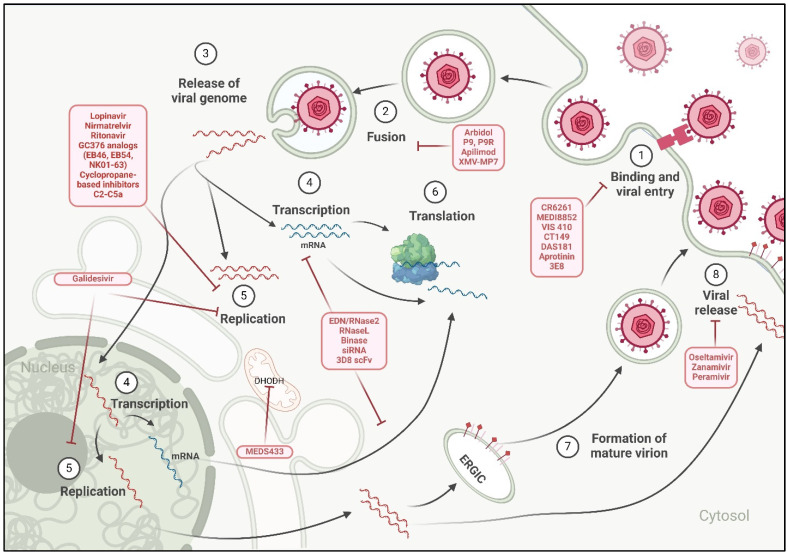
Antiviral strategies block key steps of the viral replication cycle against respiratory RNA viruses (RRVs) by broad-spectrum antiviral agents that are currently in use or under investigation. The viral life cycle involves several key steps: (1) binding and entry of the virus into the host cell, (2) membrane fusion, (3) release of the viral genome into the host cell, (4) transcription, (5) replication, (6) translation, (7) assembly of mature virions, and (8) viral release. Both cytoplasmic and nuclear viruses share most of these steps, except for transcription (step 4) and replication (step 5). For nuclear viruses, the viral genome is transported to the nucleus, where transcription and replication occur. In contrast, cytoplasmic viruses replicate and transcribe their genome within the cytoplasm. These antiviral drugs target one of each step of the viral replication cycle, viz, inhibit viral attachment, or fusion, or viral replication, or directly target the viral RNA genome, or viral mRNA steps. Created using BioRender.com.

The development of broad-spectrum antiviral agents targeting respiratory viruses holds tremendous promise in addressing the global burden of respiratory infections. By targeting conserved viral components or host factors that are crucial for viral replication, these agents provide the potential to combat a wide range of RRVs, including influenza viruses, coronaviruses, and RSVs. Diverse approaches (summarized in Figure 2 and Table 3) encompass monoclonal antibodies such as CR6261 and VIS410, focusing on the conserved regions of viral surface proteins, as well as host-directed antiviral agents such as DAS181 and umifenovir, which disrupt host cell receptors or interfere with viral replication machinery. Furthermore, viral protease inhibitors, siRNA therapies, ribonucleases, and the 3D8 scFv have demonstrated potential efficacy against various RRVs. Nonetheless, there are challenges such as drug resistance, viral evolution, and host toxicity that must be carefully addressed in the pursuit of effective therapeutics. Thus, further evaluation of host-targeted antivirals should be prioritized to minimize resistance driven by viral mutations. Studies should emphasize understanding host–pathogen interactions to refine these interventions. In addition, optimizing delivery systems to improve stability and bioavailability, especially for siRNA and ribonuclease-based therapies, is crucial. Despite these challenges, the potential broad-spectrum antiviral agents warrant further investigation and clinical verification.

The COVID-19 pandemic highlighted the critical need for rapid response frameworks. Global collaborations among researchers, clinicians, and pharmaceutical companies demonstrated the value of pooling data and resources to expedite drug evaluations. The development of Paxlovid exemplifies how partnerships between governments, academia, and industry can accelerate innovation. To sustain such progress, it is essential to establish international coalitions that provide long-term funding for broad-spectrum antiviral agents (BSAAs). Incentivizing pharmaceutical investment in antiviral R&D, alongside leveraging bioinformatics and AI, can enhance the prediction of viral evolution and guide BSAA development. Additionally, fostering data-sharing initiatives across borders will ensure the integration of preclinical and clinical findings, facilitating faster decision-making. By focusing on these strategies, the global scientific community can build a resilient framework to combat future respiratory viral threats effectively.

## Figures and Tables

**Table 2:** Representative of RRVs expressing proteolytically active proteins [125].

Virus	Family	Protease Name	Catalytic Type	Catalytic Center
Severe acute respiratory syndrome coronavirus	Coronaviridae	SARS-CoV papain-like peptidase (SARS-CoV PL^pro^)	Cysteine	Cys-His-Asp
		SARS-CoV 3CLpro (SARS-CoV M^pro^)	Cysteine	His-Cys

**Table 3:** Summary of broad-spectrum antiviral agents described in this article.

Antiviral Agents		Structure	Viruses Targeting	Mode of Action		Clinical Progress
CR6261	Monoclonal anti-HA stalk antibody	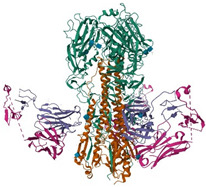 IAV HA from A/Ohio/09/2015 bound to the stalk binding CR6261 antibody Fab (PDB: 6UYN)	Influenza subtypes including H1, H2, H5, H6, H8, H9 in vitro. H1N1 and H5N1 in vivo. It has been under clinical trials [70,71,72]	Blocking the pH-induced conformational rearrangements in HA [71,72,74,75]	Direct-acting antiviral	Phase 2
MEDI8852	Monoclonal anti-HA stalk antibody	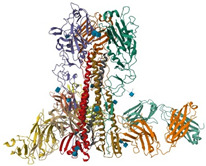 MEDI8852 Fab Fragment in complex with H5 HA (PDB: 5JW4)	H7N9 and H5N1 in vitro and in vivo with prophylaxis and therapeutic approaches, and under clinical trials [77,78]	Binding to HA [78]	Direct-acting antiviral	Phase 2
VIS410	Monoclonal anti-HA stalk antibody	-	Influenza subtypes including H1, H5, H3, H7 in vitro, in vivo, and under clinical trials [84]	Binding to the stem region of HA [79]	Direct-acting antiviral	Phase 2
CT149	Monoclonal anti-HA stalk antibody	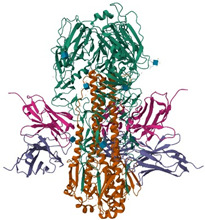 H7 HA from A/Anhui/1/2013 in complex with a neutralizing antibody CT149 (PDB: 4R8W)	Influenza subtypes including H1, H5, H3, H7 in vitro [227]	Inhibiting low pH-induced, HA-mediated membrane fusion [227]	Direct-acting antiviral	Preclinical
DAS181	A recombinant sialidase	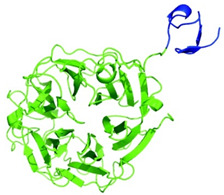 Molecular model of DAS181 [85]	H1N1, H3N2, H5N1, influenza B, PIV in vitro, in vivo, and under clinical trials [68,87,88,90]	Cleaving α2,3- and α2,6-linked sialic acid receptors on cell surface [65,68,85]	Host-directed antiviral	Phase 2, 3 (COVID 19) Phase 3 (PIV) Phase 1, 2 (influenza virus)
Umifenovir (Arbidol)	An indole-based hydrophobic molecule	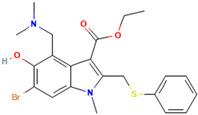 2D structure [228]	Influenza A, B, C viruses, RSV, SARS-CoV, HCoV-OC43, HCoV-229E, SARS-CoV-2, PIV-5 in vitro and/or in vivo [93,94,95,96,97]	Interacting with certain aromatic residues within the viral glycoprotein, and/or cellular proteins, resulting in blocking viral endocytosis and replication [93,94]	Direct-acting antiviral Host-directed antiviral	Phase 3, 4 (influenza virus) Phase 4 (coronavirus)
Aprotinin	A pan-protease inhibitor	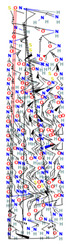 Chemical structure [229]	Influenza viruses including: H1N1, H3N2, H5N2, H6N5, H9N2, oseltamivir-resistant IAV, influenza B, SARS-CoV-2 in vitro, and/or in vivo, and under clinical trials [104,107,109,111,112]	Inhibiting proteolytic activation of some viruses [104,105,106,107]	Direct-acting antiviral Host-directed antiviral	Preclinical
3E8	A monoclonal antibody	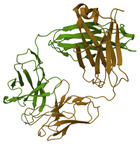 3E8 structure (from the complex of 3E8 and ACE2 structure, PDB: 7V61)	hCoV-NL63, SARS-CoV, SARS-CoV-2, and SARS-CoV-2 mutant variants (SARS-CoV-2-D614G, B.1.1.7, B.1.351, B.1.617.1, and P.1) [113].	Targeting the RBD-binding site on ACE2 [113]	Host-directed antiviral	Preclinical
PIKfyve (Apilimod, XMU-MP-7)	Fusion inhibitor	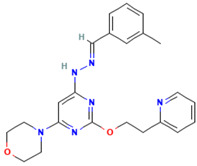 Apilimod [230]	SARS-CoV-2 and its variants (alpha, beta, delta, omicron), H1N1, H3N2, H5N1 and influenza B, and RSV in vitro and/or in vivo. Apilimod has been under clinical trials [116,118] [121]	Causing Ptdlns(3,5)P2 depletion, virus consequently is trapped within the endosomes and is unable to do fusion step [115,119]	Host-directed antiviral	Phase 2—Apilimod (COVID-19)
P9 and P9R	Peptide	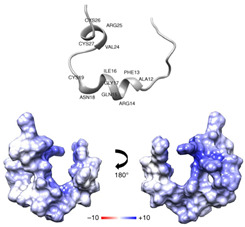 P9R peptide [122]	SARS-CoV, MERS-CoV, SARS-CoV-2, pandemic H1N1, H3N2, H5N1, H7N7, H7N9, HRV [122,123]	Binding to virus and inhibiting endosomal acidification [122]	Direct-acting antiviral Host-directed antiviral	Preclinical
Lopinavir	3CLpro inhibitor	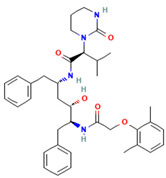 Lopinavir [231]	SARS-CoV, MERS-CoV in vitro [140,144,145,146]	Inhibiting 3CL protease	Direct-acting antiviral	Preclinical
Paxlovid (Nirmatrelvir/Ritonavir)	3CLpro inhibitor	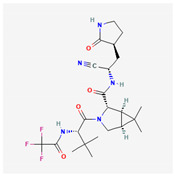 Nirmatrelvir (PubChem CID: 155903259)	SARS-CoV-2, MERS-CoV, hCoV-229E, hCoV-NL63, and hCoV-OC43 [147,148,149,150]	binding to the catalytic site cysteine, resulting in blocking the function of 3CLpro [147,148,149,150]	Direct-acting antiviral	Phase 3—Nirmatrelvir/Ritonavir (COVID-19) Paxlovid has been approval by FDA
GC376 analogs (EB46, EB54, NK01-63)	3CLpro inhibitor	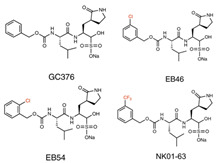 [129]	SARS-CoV, SARS-CoV -2, MERS-CoV, HCoV-229E, HCoV-OC43 in vitro [129]	Inhibiting 3CL protease	Direct-acting antiviral	Preclinical
Cyclopropane-based inhibitors	3CLpro inhibitor	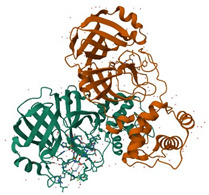 SARS-CoV-2 3CL protease in complex with the cyclopropane based inhibitor 5c (PDB: 7TQ3)	MERS-CoV, SARS-CoV, SARS-CoV-2 in vitro [128]	Inhibiting 3CL protease	Direct-acting antiviral	Preclinical
C2-C5a	3CLpro inhibitor	-	Omicron subvariants (BA.5, BQ.1.1, and XBB.1.5) and HCoV-229E in vitro [153]	Inhibiting 3CL protease	Direct-acting antiviral	Preclinical
Galidesivir	a RdRp inhibitor	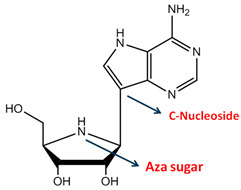 Structure of Galidesivir [154]	MERS-CoV, SARS-CoV, IAV, RSV, and HRV in vitro, and under clinical trials [154,155,156]	Mimic the natural nucleoside agents, RdRp recruits the artificial nucleotides, resulting in being unable to add more nucleotides [154,155]	Direct-acting antiviral	Phase 1 (COVID-19)
MEDS433	hDHODH inhibitor	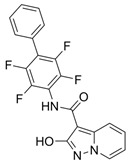 Structure of MEDS433 [161]	influenza A, B viruses, RSV, HCoV-299E, OC43, and SARS-CoV-2 [54,161,162]	Inhibiting enzyme hDHODH, resulting in suppressing pyrimidine biosynthesis pathway	Host-directed antiviral	Preclinical
EDN/RNases2	Ribonuclease	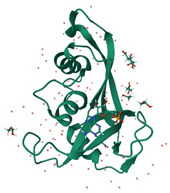 EDN/ribonuclease2 in complex with 5′-adenosine monophosphate (AMP) (PDB: 8F5X)	RSV-B, and PIV in vitro [165,166]	Ribonuclease-dependent activity [165,166]	Direct-acting antiviral	Preclinical
RNase L	Ribonuclease	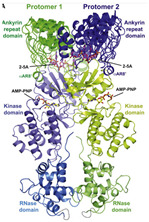 Structure of RNaseL dimer [232]	IAV in absence of viral resistance NS1 protein and RSV in IFN-γ-mediated inhibition in vitro [167,168]	Ribonuclease-dependent activity, IFN activation [167,168]	Direct-acting antiviral Host-directed antiviral	Preclinical
Binase	Ribonuclease	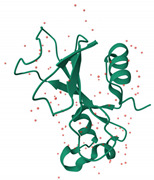 Ribonuclease Binase (G specific endonuclease) unliganded form (PDB:1GOU)	MERS-CoV, HCoV-229E, H1N1pdm09 and HRV1A in vitro [170,171]	ribonuclease-dependent activity [170,171]	Direct-acting antiviral	Preclinical
siRNA	RNAi	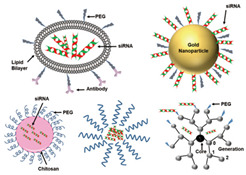 Nanoparticle and polymer complex based siRNA carriers systems [233]	SARS-CoV-2 variants, SARS-CoV, MERS-CoV, RSV, PIV, influenza virus in vitro and/or in vivo and/or ex vivo [173,174,175,176,177,178,179,180,181]	Digesting viral genome	Direct-acting antiviral Host-directed antiviral	Preclinical
3D8 scFv	a monoclonal antibody	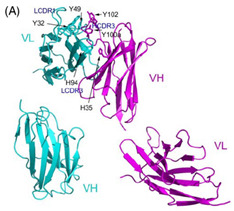 Structure of 3D8 scFv and VH, VL [199]	H1N1/PR8, H1N1/NWS33, H1N1/pdm09, H9N2, H3N2; coronaviruses: HCoV-OC43, SARS-CoV-2, PEDV in vitro and/or in vivo [23,39,200,201,207]	May digest viral genome based on nucleic acid-hydrolyzing activity	Direct-acting antiviral	Preclinical
Oseltamivir	Neuramiridase inhibitor	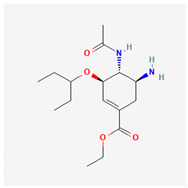 Chemical structure of Oseltamivir (PubChem CID: 60528)	Influenza A viruses, influenza B viruses [211]	Blocking the NA active site of influenza virus, impairing virus release and effectively limiting reinfection [209]	Direct-acting antiviral	Approved
Zanamivir	Neuramiridase inhibitor	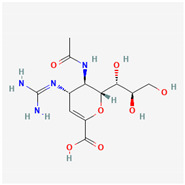 Chemical structure of Zanamivir (PubChem CID: 60855)	Influenza A viruses, influenza B viruses [211]	Blocking the NA active site of influenza virus, impairing virus release and effectively limiting reinfection [209]	Direct-acting antiviral	Approved
Peramivir	Neuramiridase inhibitor	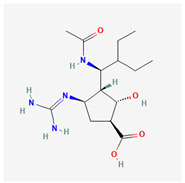 Chemical structure of Peramivir (PubChem CID: 154234)	Influenza A viruses, influenza B viruses [211]	Blocking the NA active site of influenza virus, impairing virus release and effectively limiting reinfection [209]	Direct-acting antiviral	Approved
